# Solid-Phase Synthesis of Arylpiperazine Derivatives and Implementation of the Distributed Drug Discovery (D3) Project in the Search for CNS Agents

**DOI:** 10.3390/molecules16054104

**Published:** 2011-05-19

**Authors:** Paweł Zajdel, Joanna Król, Katarzyna Grychowska, Maciej Pawłowski, Gilles Subra, Gaël Nomezine, Jean Martinez, Grzegorz Satała, Andrzej J. Bojarski, Ziniu Zhou, Martin J. O’Donnell, William L. Scott

**Affiliations:** 1 Department of Medicinal Chemistry, Jagiellonian University Medical College, 9 Medyczna Street, Kraków 30-688, Poland; 2 Équipe Aminoacides Peptides et Protéines, Institut des Biomolécules Max Mousseron IBMM, UMR CNRS 5247, Faculté de Pharmacie, Université Montpellier I et II, 15 avenue Charles Flahault, Montpellier 34060, France; 3 Department of Medicinal Chemistry, Institute of Pharmacology, Polish Academy of Sciences,12 Smętna Street, Kraków 31-343, Poland; 4 Department of Chemistry and Chemical Biology, Indiana University-Purdue University Indianapolis, Indianapolis, IN 46202, USA

**Keywords:** Distributed Drug Discovery (D3), combinatorial chemistry, solid-phase synthesis, SynPhase Lanterns, long-chain arylpiperazines, succinimides, 5-HT_1A_, 5-HT_2A_ receptor affinity

## Abstract

We have successfully implemented the concept of Distributed Drug Discovery (D3) in the search for CNS agents. Herein, we demonstrate, for the first time, student engagement from different sites around the globe in the development of new biologically active compounds. As an outcome we have synthesized a 24-membered library of arylpiperazine derivatives targeted to 5-HT_1A_ and 5-HT_2A_ receptors. The synthesis was simultaneously performed on BAL-MBHA-PS resin in Poland and the United States, and on BAL-PS-SynPhase Lanterns in France. The D3 project strategy opens the possibility of obtaining potent 5-HT_1A_/5-HT_2A_ agents in a distributed fashion. While the biological testing is still centralized, this combination of distributed synthesis with screening will enable a D3 network of students world-wide to participate, as part of their education, in the synthesis and testing of this class of biologically active compounds.

## Abbreviations

CHCl_3_: chloroform
DCM: dichloromethane
DIC: *N,N*’-diisopropylcarbodiimide
DIEA: diisopropylethylamine
DMF: dimethylformamide
HBTU: *O*-(1*H*-benzotriazol-1-yl)-1,1,3,3-tetramethyluronium hexafluorophosphate
SOCl_2_: thionyl chloride
TFA: trifluoroacetic acid

## 1. Introduction

**Figure 1:**
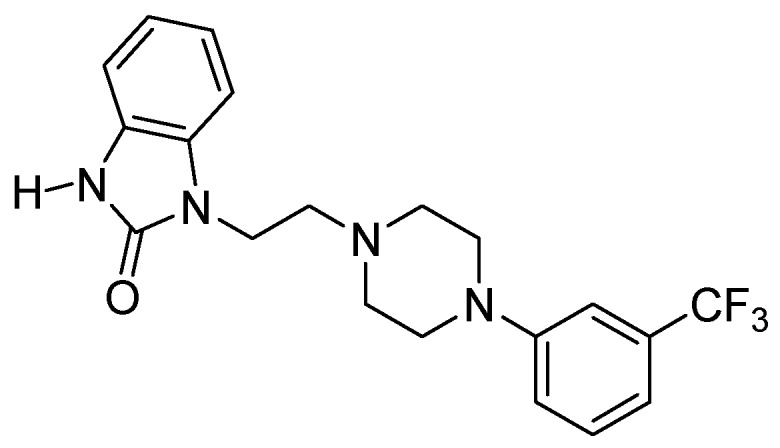
Chemical structure of flibanserin.

Arylpiperazines are valuable investigative tools in neuropsychopharmacology and have the potential to be leads in the development of new therapeutic agents. Flibanserin (Figure 1) is an excellent example of a classic long-chain arylpiperazine with an interesting new application. This *meta*-trifluorophenylpiperazine derivative, classified as a 5-HT_1A_ agonist and 5-HT_2A_ antagonist, had initially been developed as an antidepressant [1,2]. Later it was found that it exerts potential antipsychotic effects. It has also been investigated as a drug for women with decreased sexual desire [3].

**Figure 2:**
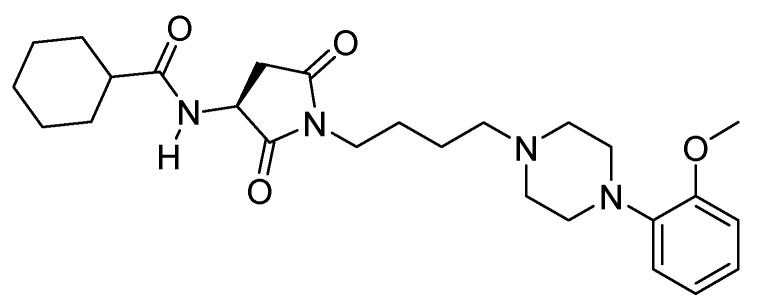
Chemical structure of PZ-68.

Previously we demonstrated that a member of an arylpiperazine library containing a cyclized *N*-acylated aspartic acid (PZ-68, Figure 2), was a partial agonist of 5-HT_1A_ receptors and a 5-HT_2A_ antagonist, and showed *in vivo* antidepressant-like activity at doses of 5–20 mg/kg [4].

**Figure 3:**
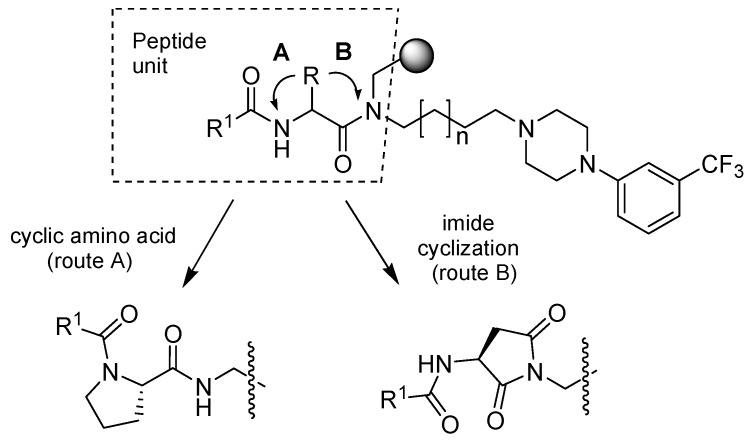
General structure of the target compounds **A** and **B** obtained via modification of the peptide unit 1.

It is well known [5,6,7] that the affinity profile, functional activity and therapeutic potential (mostly antipsychotic, antidepressant, and/or anxiolytic) of arylpiperazine derivatives depends on the substitution pattern of the phenyl ring, linker length, and the terminal substructures. Accordingly, we designed a new series of *meta*-trifluoromethyl phenylpiperazine analogs of the most interesting previous sets containing *N*-acyl-3-aminopyrrolidine-2,5-diones and *N*-acylprolinamides. These molecules involved conformational restriction of a peptide unit in two ways: introduction of a cyclic amino acid (scaffold **A**, Figure 3), or an imide (scaffold **B**, Figure 3, resulting from an internal cyclization of aspartic acid). Based on our previous results [8], the structural modifications also varied both the linker length (three and four methylene groups) between the amide and the basic nitrogen atom of the arylpiperazine fragment, and the R^2^ acyl-substituents.

This work provided an opportunity to further enable the concept of Distributed Drug Discovery (D3) [9,10,11] by combining education, synthesis and biological screening. The chemistry required for SAR evaluation can be reproducibly carried out at distant sites, using simple and inexpensive equipment. Adding biological testing, even if centralized, with distributed synthesis engages students in the D3 network in these two key disciplines of drug discovery.

## 2. Results

### 2.1. Library Synthesis

**Scheme 1:**
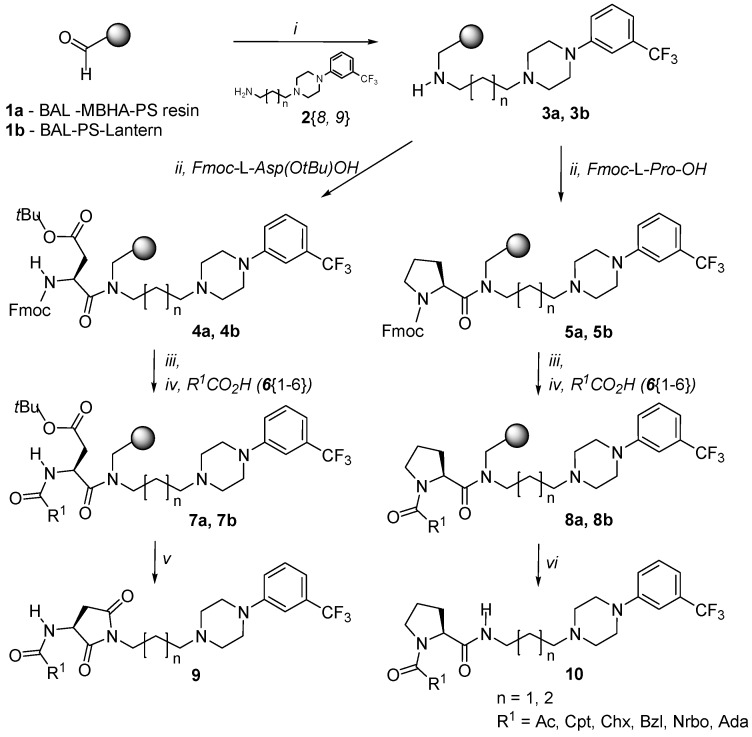
Synthetic route to *N*-acyl-3-aminopyrrolidine-2,5-diones and *N*-acylprolinamides: (*i*) amine diversity reagents **2**{*8, 9*} [14], NaBH_3_CN, 1% AcOH/DMF, rt, 24 h; (*ii*) DIC, DMF, rt, 12 h, 2×; (*iii*) 20% piperidine/DMF (*iv*) Diversity reagents **6**{*1–6*}, HBTU, DIEA, DMF, RT, 3 h (series **a**) or 2 h (series **b**); (*v*) TFA/CHCl_3_/SOCl_2_, 35 °C, 12 h (series **a**) or 40 °C, 10 h (series **b**); (*vi*) TFA/CH_2_Cl_2_, RT, 1.5 h (series **a**) or 1 h (series **b**). Synthesis of series **a** was carried out on BAL-MBHA-PS resin, while series **b** was synthesized on BAL-PS-Lantern. Chemset numbering (Figure 4 and Figure 5) corresponds to the system applied in the historical libraries [9,14,15].

A parallel solid-phase synthesis was carried out on two types of solid supports: BAL linker functionalized *p*-methylbenzhydrylamine (MBHA) polystyrene resin (**1a**) and BAL linker functionalized polystyrene SynPhase Lanterns (**1b**, Scheme 1).

The solid-phase chemistry on MBHA resin was adapted from the protocol reported on SynPhase Lanterns [9]. The separate, multiple syntheses on the resin were performed manually by using a Bill-Board set [10,11,12,13]. This equipment keeps the solid-phase reactions organized in a grid and simplifies repeated cycles of reactions, washing, cleavage, and the final solvent evaporation step. One compound was synthesized in each of the reaction vessels, which were organized on a solvent resistant 2 × 3 grid.

**Figure 4:**
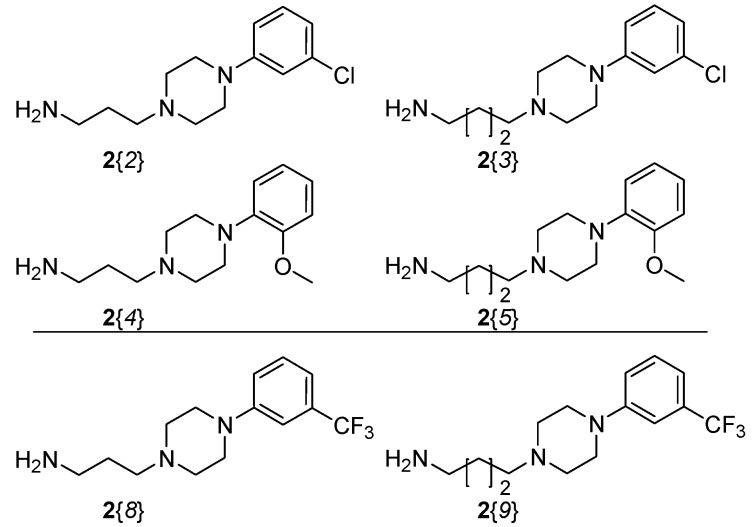
Diversity reagent **2**. Amines **2**{*8-9*} [14] used in the current project. Amines **2**{*2–5*} were used to synthesize historical compounds [9,15] presented for comparison in the radioligand binding experiments.

The syntheses began with the preparation of 4-(3-trifluoromethylphenyl)-1-piperazinylpropyl- and butylamine (**2**{*8*} and **2**{*9*}, Figure 4).

**Figure 5:**
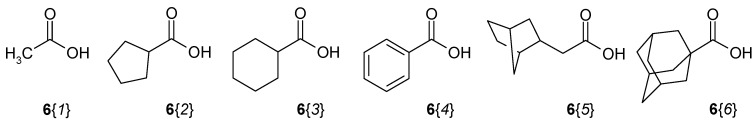
Diversity of carboxylic acids **6**{*1–6*}.

These amines were attached to the resin by a one-pot reductive amination to give the support-bound secondary amine products **3**. Fmoc-protected amino acids were then coupled to the support by a symmetric anhydride method using DIC in DMF to give chemsets **4** and **5**. Following Fmoc removal, the amines were coupled with carboxylic acids (diversity reagents **6**, Figure 5) in the presence of HBTU under basic conditions. The final pyrrolidine-2,5-dione derivatives **9** were obtained in a one-pot cleavage-cyclization step using a mixture of TFA/CHCl_3_/SOCl_2_, while prolinamides **10** were obtained directly after treatment with TFA/CH_2_Cl_2_.

In parallel to solid-phase chemistry on the resin we have performed syntheses of selected library representatives on SynPhase Lanterns. This modular solid-support consists of soluble polymers grafted onto a rigid unreactive base polymer. As a tagging system for SynPhase Lanterns, we have used colored cogs and spindles (corresponding to the building block). At each step the Lanterns were manually sorted and pooled into separate vials containing respective reagents. Cleavage of the final products was performed in glass vials. The syntheses were conducted in standard laboratory glassware according to the protocol previously reported [9]. The crude purities of selected compounds synthesized in parallel on resin and Lanterns were determined and compared after removal of the cleavage cocktail (Table 1).

**Table 1:** Comparison of the analytical data for selected library members obtained from either resin or Lanterns.

Compound	Purity ^a^		Compound	Purity ^a^	
	Lantern	Resin		Lantern	Resin
**9**{*8,3*}	74	61	**10**{*8,3*}	96	93
**9**{*8,4*}	70	52	**10**{*8,4*}	94	93
**9**{*8,5*}	77	59	**10**{*8,5*}	96	87
**9**{*9,3*}	68	62	**10**{*9,3*}	97	97
**9**{*9,4*}	64	49	**10**{*9,4*}	98	89
**9**{*9,6*}	75	56	**10**{*9,6*}	98	86

^a^ Percent purity of the crude product was calculated on the peak area integration during HPLC analysis of cleaved compounds at a sum of wavelengths between 200 to 270 nm.

All the compounds synthesized on the BAL-MBHA-PS resin were purified on silica gel (now using the reactor vessels to contain the silica for column chromatography) using DCM/MeOH (9/1 or 9/0.7, v/v) mixture. The percent purity was based on the peak area integration during HPLC analysis of cleaved compounds at a sum of wavelengths between 200 to 270 nm (Table 2).

**Table 2:** Analytical data of the library.

Compound	R_t_ (min)	Purity ^a^	MW calc.	[M + H]^+^ found	Compd	R_t_ (min)	Purity^a^	MW calc.	[M + H]^+^ found
**9**{*8,1*}	1.35	97	426.2	427.2	**10**{*8,1*}	1.34	99	426.2	427.3
**9**{*8,2*}	1.60	95	480.2	481.4	**10**{*8,2*}	1.62	99	480.3	481.0
**9**{*8,3*}	1.66	95	494.2	495.1	**10**{*8,3*}	1.69	96	494.3	495.2
**9**{*8,4*}	1.60	95	488.2	489.3	**10**{*8,4*}	1.58	96	488.2	489.8
**9**{*8,5*}	1.78	95	520.2	521.6	**10**{*8,5*}	1.82	96	520.3	521.5
**9**{*8,6*}	1.85	99	546.3	547.4	**10**{*8,6*}	1.90	97	546.3	547.4
**9**{*9,1*}	1.38	97	440.2	441.5	**10**{*9,1*}	1.37	93	440.2	441.5
**9**{*9,2*}	1.60	99	494.2	495.5	**10**{*9,2*}	1.60	96	494.3	495.1
**9**{*9,3*}	1.67	99	508.2	509.3	**10**{*9,3*}	1.68	97	508.3	509.3
**9**{*9,4*}	1.62	95	502.2	503.4	**10**{*9,4*}	1.58	97	502.2	503.3
**9**{*9,5*}	1.78	96	534.3	535.4	**10**{*9,5*}	1.80	98	534.3	535.6
**9**{*9,6*}	1.85	98	560.3	561.4	**10**{*9,6*}	1.86	96	560.3	561.1

^a^ Percent purity of the purified product was calculated on the peak area integration during HPLC analysis of cleaved compounds at a sum of wavelengths between 200 to 270 nm.

### 2.2. Biological Evaluation

The affinity for serotonin 5-HT_1A_ and 5-HT_2A_ receptors of 13 selected library representatives was measured *in vitro* (Table 3, column 1) on the basis of the screening protocol described previously [9]. At the same time, the two well-known reference serotonin drugs buspirone and clozapine were examined, the obtained results being consistent with our previous data as well as with those reported in the literature (Table 3) [8].

**Table 3:** Radioligand binding data of synthesized compounds for 5-HT_1A_ and 5-HT_2A_.

Compound *m*-CF_3_ set	*K*_i_ [nM] ^a^		Compound *m*-Cl set ^b^	*K*_i_ [nM]		Compd *o*-OCH_3_ set ^b^	*K*_i_ [nM]	
	5-HT_1A_	5-HT_2A_		5-HT_1A_	5-HT_2A_		5-HT_1A_	5-HT_2A_
**9**{*8,1*}	129	164	**9**{*2,1*} ^c^	NT ^e^	NT	**9**{*4,1*} ^d^	480	NT
**9**{*8,3*}	170	276	**9**{*2,3*} ^c^	360	92	**9**{*4,3*} ^d^	230	NT
**9**{*9,2*}	24	79	**9**{*3,2*}	28	47	**9**{*5,2*}	23	480
**9**{*9,3*}	19	70	**9**{*3,3*}	26	6.5	**9**{*5,3*}	19	183
**9**{*9,4*}	18	61	**9**{*3,4*}	52	20	**9**{*5,4*}	18	410
**9**{*9,5*}	15	30	**9**{*3,5*}	21	4.2	**9**{*5,5*}	9	47
**9**{*9,6*}	21	39	**9**{*3,6*}	47	25	**9**{*5,6*}	4	173
**10**{*8,4*} ^f^	122	898	**10**{*2,4*}	NT	875	**10**{*4,4*}	NT	NT
**10**{*8,6*} ^f^	42	1651	**10**{2*,6*}	75	360	**10**{*4,6*}	NT	NT
**10**{*9,2*} ^f^	26	191	**10**{*3,2*} ^c^	NT	NT	**10**{*5,2*}	NT	NT
**10**{*9,4*}^ f^	19	145	**10**{*3,4*}	78	128	**10**{*5,4*}	NT	NT
**10**{*9,5*} ^f^	10	82	**10**{*3,5*}	37	35	**10**{*5,5*}	14	547
**10**{*9,6*} ^f^	8	699	**10**{*3,6*}	13	140	**10**{*5,6*}	3	503

^a^ Estimated *K*_i_ values; as the reference drugs buspirone (*K*_i_(5-HT_1A_) = 36 nM) and clozapine (*K*_i_(5-HT_2A_) = 6.05 nM) we used; ^b^ Data taken from references [4] if not otherwise stated; ^c^ Data taken from reference [9]; ^d^ Data taken from reference [15]; ^e^ NT = Not tested; ^f^ Proline amides from chemset **10** correspond to chemset **17** from historical libraries [9,15].

## 3. Discussion

The D3 program, developed at IUPUI to engage students in the discovery of new biologically active chemical entities, provided a framework to create a small international working group. The ultimate goal of the D3 project is to discover compounds for neglected diseases prevalent in developing countries. We wanted to demonstrate the successful application of the D3 process in the design, synthesis and biological evaluation, against 5-HT receptors, of this small library of arylpiperazine derivatives. The library design was based on our previously successful introduction of *N*-acylated amino acids fragments into the arylpiperazine moiety yielding potent 5-HT_1A_ and 5-HT_2A_ receptor ligands [8,9,15,16]. For the educational purpose, in the current project we chose a subset of analogs of the most interesting sets containing *N*-acyl-3-aminopyrrolidine-2,5-diones and *N*-acylprolinamides. We wished to enable students to further continue the structural modifications in the arylpiperazine fragment and differentiate the length of the alkyl spacer.

The library synthesis strategy was based on the previously developed solid-phase methodology performed on SynPhase Lanterns [9]. For the purpose of the D3 project we adapted the Lantern protocol to MBHA resin. Synthesis of the library on the resin beads was performed in Krakow and Indianapolis, while the modular Lantern solid supports were used in Montpellier. Average overall yields of the crude products, calculated on the basis of the initial loading of the solid support, were 50% to 69% and 35% to 58% for the resin and Lanterns, respectively. The crude purities of the compounds differed slightly among the laboratories. For the identified library members synthesized on the Lanterns the average purity was 76%, while for compounds obtained from the resin the average purity was 69%. Generally, Lanterns provided products with higher crude purities than the resin (Table 1). Within the series, the compounds bearing a proline residue (chemset **10**) were always of higher purity. This is likely a function of the simple cleavage from the linker involving TFA.

To fulfill the quality standards for the combinatorial libraries and demonstrate compatibility with the D3 process, all 24 members were purified using the synthesis vessels now filled with silica gel as simple and inexpensive chromatography columns (Table 2). This enabled us to perform a distributed synthesis and to obtain replicated results by students from three sites around the globe: Jagiellonian University Medical College (Poland), University Montpellier, Biomolecules Institute Max Mousseron (France) and Indiana University-Purdue University Indianapolis (USA).

For the next stage of the project we selected 13 library representatives and tested them for their affinity for 5-HT_1A_ and 5-HT_2A_ receptors. Biological evaluation was also performed by students in the Polish Academy of Science. This is the first presentation of our synthetic cross-boundary efforts followed by biological evaluation of the new compounds within D3 project. The investigated compounds displayed low-to-good affinity for 5-HT_2A_ receptors (*K*_i_ = 1651 to 30 nM) and good-to-high affinity for 5-HT_1A_ receptors (*K*_i_ = 170 to 8 nM) in *in vitro* binding experiments. Generally, compounds containing a four methylene spacer were more potent 5-HT_1A_ agents than their propylene counterparts, e.g., **9**{*8,3*} *vs.*** 9**{*9,3*} and **10**{*8,4*} *vs.* **10**{*9,4*}.

To continue the structure-activity relationship studies we compared the binding data of the new compounds (Table 3, column 1) with the historical data for the *meta*-chloro [4,9] and *ortho*-methoxy [4,15] analogs (Table 3, column 2 and 3). Replacement of the chlorine atom in the *meta* position of the phenyl ring with the more bulky trifluoromethyl substituent increased affinity of the new compounds for 5-HT_1A_ receptors. Interestingly, the 5-HT_1A_ receptor affinity of the compounds bearing a *meta*-CF_3_ substituent was comparable to the affinity of *ortho*-OCH_3_ derivatives (well-known 5-HT_1A_ ligands). On the other hand, this modification decreased affinity of the new compounds for 5-HT_2A_ receptors in comparison to their *meta*-chloro analogs. In opposite to *ortho*-OCH_3_ substituted pyrrolidine-2,5-dione derivatives, their *meta*-substituted analogs (Cl and CF_3_) presented limited dependence of volume of cycloalkylcarbonyl fragment on 5-HT_1A_ receptor affinity; on the other hand, 5-HT_1A_ receptor affinity of *meta*-CF_3_ prolinamides was clearly dependent on the kind of cycloalkyl substituent. It is worth noting that compounds containing the norborn-2-yl moiety in the cycloalkyl fragment were always the most potent 5-HT_2A_ agents.

## 4. Experimental

### 4.1. General Methods

Solution and solid-phase organic transformations, Lanterns and resin washes were carried out at ambient temperature, unless indicated otherwise. Organic solvents (from Acros Organics, Aldrich) were of reagent grade and were used without purification. PS-BAL linker SynPhase Lanterns with 38-μmol loading were purchased from Mimotopes Pty, Australia. 4-Methylbenzhydrylamine hydrochloride resin (PL-MBHA·HCl, 1.6 mmol/g, 75–150 μm) was purchased from Polymer Laboratories. 4-(4-Formyl-3,5-dimethoxyphenoxy) butyric acid (BAL linker) was purchased from NovaBiochem. All the Fmoc amino acids and HBTU reagent were purchased from NovaBiochem and from Iris Chemicals. All other reagents were from Aldrich.

Purity of the synthesized compounds was confirmed by TLC performed on Merck silica gel 60 F_254_ aluminium sheets (Merck, Darmstadt, Germany). Spots were detected by their absorption under UV light (λ = 254 nm). In all the cases HPLC technique was used as a routine.

Analytical HPLC were run on a Waters Alliance HPLC instrument, equipped with a Chromolith SpeedROD column (4.6 × 50 mm). Standard conditions were eluent system A (water/0.1% TFA), system B (acetonitrile/0.1% TFA). A flow rate of 5 mL/min and a gradient of (0–100)% B over 3 min were used. Detection was performed on a PDA detector. Retention times (*t*_R_) are given in minutes.

Column chromatography was performed on silica gel (irregular particles 40–63 μm) filled synthesis reactors. The yields of the final compounds, after chromatographic purification, were calculated on the basis of the initial loading of the starting resins and are the overall yields of all reaction steps starting from these resins.

^1^H-NMR and ^13^C-NMR spectra were recorded at 500 MHz (Bruker Avance III 500) or 300 MHz (Varian BB 200) spectrometer using TMS (0.00 ppm) and chloroform-*d*_1_, or chloroform-*d*_1_ mixed with methanol-*d*_4_ (2%–10%); *J* values are in Hertz (Hz), and splitting patterns are designated as follows: s (singlet), d (doublet), t (triplet), m (multiplet).

Electrospray ionization mass spectrometry was conducted using a PESciex API III triple stage quadrupole mass spectrometer or Waters Alliance 2690 HPLC, coupled to a Micromass (Manchester, UK) Platform II spectrometer (electrospray ionization mode) operated in either positive-ion or negative-ion detection mode. Samples were prepared in acetonitrile/water (50/50 v/v), containing a 0.1% TFA. All the analyses were carried out using a C18 Xterra MS, 2.1 × 30 mm column.

A flow rate of 500 μL/min and a gradient of (0–100)% B over 5 min were used. Eluent A: water/0.1% TFA; eluent B: acetonitrile/0.1% TFA. Positive ion electrospray mass spectra were acquired at a solvent flow rate of 100–500 μL/min. Nitrogen was used for both the nebulizing gas and the drying gas. The data were obtained in a scan mode ranging from 400 to 1400 *m/z* in 0.1 s intervals; 10 scans were summed up to get the final spectrum.

### 4.2. General Procedures for Manual Solid-Phase Reactions

Manual solid-phase organic syntheses at ambient temperature were carried out in several types of reaction vessels: 50 mL peptide synthesis reaction vessels with coarse porosity fritted glass support and supplied with a GL thread and a Teflon lined PBT screw cap (ChemGlass, CG-1860-03) were used for large scale (up to 3.7 mmol) reactions. Small scale reactions (typically 50 μmol) were performed in 3.5 mL fritted glass reaction vessels equipped with polypropylene screw caps with Teflon faced silicon septa on the Bill-Board set [13], which was designed by one of us (WLS) as inexpensive equipment to simplify and expedite multiple, manual solid-phase syntheses. The Bill-Board reaction vessel components are available from Chem-Glass: IUP-0305-270H for 3.5 mL reaction vessel; IUP-0305-280H (polypropylene screw cap); CV-4080-0013 (Teflon faced silicone septa).

For agitation purpose, the large scale reactions in the peptide synthesizers were placed on an orbital shaker Roto Mix while motor rotators were used for small scale reactions. Resin-bound intermediates were air-dried after the final CH_2_Cl_2_ washes, unless they needed to be re-weighed in which case overnight drying under high vacuum (≤2 mm) or under low vacuum over 24–36 h in a vacuum desiccator was carried out. During wash of the resin with solvents in the reaction vessels, at least 3 min of gravity draining was used for large scale whereas at least 30 seconds is needed for small scale followed by air-push.

BAL linker polystyrene SynPhase Lanterns with 38-μmol loading, and colored tagging system were from Mimotopes (Clayton, Australia) [17]. To manually manage the library construction, a split-and-pool approach was chosen. The Lanterns were equipped with colored cogs and spindles (corresponding to building blocks) producing a visual tagging system. Solid-phase manipulations involving SynPhase Lanterns were carried out in a glass reaction vessels capped with silicone caps. As a standard the Lanterns were washed with respective solvents by immersing them in DMF (3 × 5 min), MeOH (1 × 5 min), and CH_2_Cl_2_ (3 × 5 min), respectively. A single 200-mL standard Schott flask equipped with a drilled topper was used. The Lanterns were allowed to air-dry for 15 min after the last CH_2_Cl_2_ washing.

### 4.3. Solid-Phase Synthesis on BAL-MBHA-PS Resin

*Preparation of BAL-MBHA-PS Resin* (**1a***,* Scheme 1): MBHA HCl-PS resin (0.469 g, 0.75 mmol, 1.6 mmol/g) loaded in a 25 mL peptide synthesis reaction vessel was sequentially washed with DMF (3 × 8 mL), CH_2_Cl_2_ (2 × 8 mL), 10% DIEA/ CH_2_Cl_2_ (5 × 8 mL) and CH_2_Cl_2_ (2 × 8 mL). A solution of 4-(4-formyl-3,5-dimethoxyphenoxy)butyric acid (0.805 g, 3.00 mmol, 4 equiv.), HBTU (1.138 g, 3.00 mmol, 4 equiv.) and DIEA (1.05 mL, 6.00 mmol, 8 equiv.) in anhydrous DMF prepared 5 min before was then added to the washed resin. The reaction vessel was put on the orbital shaker and allowed to shake at room temperature for 18 h. The completion of the reaction was monitored by Chloranil test. The resultant orange yellow MBHA-BAL-PS resin (**1a**) was washed with DMF (3 × 8 mL), CH_2_Cl_2_ (4 × 8 mL), and dried under low vacuum for 24 h.

#### 4.3.1. Procedure for preparation of amine-bound Resin **3a**, **3b** via reductive amination

The resin (0.9 g, 1.44 mmol) was divided into two reactors containing a suspension of sodium cyanoborohydride ([NaBH_3_CN] = 0.23 g, 3.6 mmol, 5 equiv.) and the amine ([Diversity reagent **2**, **2**{*8,9*}] = 3.6 mmol, 5 equiv., Figure 1), in a 1% acetic acid in 20 mL of DMF. The reactors were shaken on for 24 hours at room temperature. Then the resin was drained. The resin was first washed with 10% AcOH in DMF (1 × 5 mL) then with DMF (3 × 8 mL) and CH_2_Cl_2_ (4 × 8 mL), and dried under low vacuum.

#### 4.3.2. Coupling of amine resin **3a**, **3b** with *N*-α-Fmoc-Asp(OtBu)-OH and *N*-α-Fmoc-proline

Two DMF solutions (24 mL) containing a Fmoc-protected amino acid and DIC each, were freshly prepared in a standard Schott flask before acylation ([Fmoc-AA-OH] = 3.6 mmol, 5 equiv, [DIC] = 1.8 mmol, 2.5 equiv.), and the mixture was left for 15 min to form an active anhydride. Then 2 mL of the preactivated solutions were added to the resin and shaken for 12 h at a room temperature. The resin was drained and was washed following with DMF (3 × 8 mL) and CH_2_Cl_2_ (4 × 8 mL), and dried under low vacuum. The acylation was repeated one more time for 12 h.

#### 4.3.3. Standard Fmoc-deprotection protocol

The Fmoc-deprotection step was carried out by treating the resin with a mixture of piperidine and DMF (20/80; v/v) for 3 min. Then the resin was drained and the piperidine treatment was repeated for next 15 minutes. After removal of the deprotection solution, the Fmoc-deprotected resins were washed with DMF (3 × 8 mL) and CH_2_Cl_2_ (4 × 8 mL), and dried under low vacuum for 12 h.

#### 4.3.4. Acylation with carboxylic acids using HBTU

The resins **4, 5** (31mg, 0.05 mmol) were swelled in DCM (5 mL) for 20 min, and then washed with DCM/DMF (80/20; v/v). A solution of respective carboxylic acid [R^2^-COOH] = 0.25 mmol, 5 equiv. (Figure 5: Diversity reagent **6**{*1*–*6*}) was then added to the reactor, followed by HBTU (0.25 mmol, 5 equiv.) and DIEA (0.5 mmol, 10 equiv.). The solutions were added to the resin and the reactors were shaken for 3 h at room temperature. The resins were drained, and were washed with DMF (3 × 8 mL), CH_2_Cl_2_ (4 × 8 mL), and dried under low vacuum for 24 h. The procedure described above was repeated.

#### 4.3.5. Succinimide derivatives formation via cleavage and intramolecular ring closure

Supported chemsets **7a, 7b** were placed in glass vials containing a 2 mL mixture of TFA/CHCl_3_/ SOCl_2_ (50/50/1.5, v/v/v). The reaction was allowed to stand for 12 h at 35 °C. Then the solutions were filtered through the cotton via a Pasteur pipette to the evaporation vials fitted on Bill-Board. The “cocktail” was then evaporated by nitrogen flow used with Bill-Board evaporator.

#### 4.3.6. Cleavage of the proline derivatives off the resin

A 500 μL of the TFA/DCM (80/20; v/v) was dispensed into glass vials containing the resin **8a**, **8b**. Cleavage was carried out at room temperature for 90 min. The cleavage cocktail was removed from the Bill-Board glass vials under nitrogen flow.

### 4.4. Analytical Data for Biologically Tested Library Members Synthesized on MBHA Resin

*N-((S)-1-{3-[4-((3-trifluoromethyl)phenyl)piperazin-1-yl]propyl}pyrrolidin-2,5-dion-yl)acetamide* [**9**{*8,1*}]. Yield: 13 mg (59% isolated yield) as a yellow oil following chromatographic purification over silica gel with CH_2_Cl_2_/MeOH (9:1); initial LC/MS purity 73%, *t*_R_ = 1.35 min. ^1^H-NMR (300 MHz, CDCl_3_): δ 1.42–1.54 (m, 2H), 2.04 (s, 3H), 2.61–2.69 (dd, *J* = 18.5 Hz, *J* = 4.9 Hz, 1H), 2.89 (m, 2H), 3.06–3.19 (m, 1H), 3.24–3.39 (m, 4H), 3.56–3.75 (m, 6H), 4.83–4.88 (m, 1H), 7.05–7.09 (d, *J* = 8.2 Hz, 1H), 7.11 (s, 1H), 7.21–7.25 (d, *J* = 7.7 Hz, 1H), 7.38–7.44 (t, *J* = 7.9 Hz, 1H), 7.49–7.51 (d, *J* = 7.9 Hz, 1H). MS calcd for [M + H]^+^: C_20_H_26_N_4_O_3_F_3_ *m/z* 427.2, found 427.2.

*N-((S)-1-{3-[4-((3-trifluoromethyl)phenyl)piperazin-1-yl]propyl}pyrrolidin-2,5-dion-yl)cyclohexanecarboxamide* [**9**{*8,3*}]. Yield: 15 mg (61% isolated yield) as a yellow oil following chromatographic purification over silica gel with CH_2_Cl_2_/MeOH (90:7); initial LC/MS purity 61%, *t*_R_ = 1.66 min. ^1^H-NMR (300 MHz, CDCl_3_): δ 1.42–1.54 (cluster, 12H), 2.01–2.09 (m, 1H), 2.13–2.18 (m, 2H), 2.61–2.68 (dd, *J* = 18.2 Hz, *J* = 4.9 Hz, 1H), 3.06–3.12 (m, 1H), 3.27–3.36 (m, 4H), 3.56–3.71 (m, 6H), 4.72–4.79 (m, 1H), 7.06–7.09 (d, *J* = 7.9 Hz, 1H), 7.12 (s, 1H), 7.14–7.16 (d, 1H), 7.20–7.23 (d, *J* = 7.7 Hz, 1H), 7.38–7.44 (t, *J* = 7.9 Hz, 1H). MS calcd for [M + H]^+^: C_25_H_34_N_4_O_3_F_3_* m/z* 495.2, found 495.1.

*N-((S)-1-{4-[4-((3-trifluoromethyl)phenyl)piperazin-1-yl]butyl}pyrrolidin-2,5-dion-yl)cyclopentanecarboxamide* [**9**{*9,2*}]. Yield: 17 mg (69% isolated yield) as a yellow oil following chromatographic purification over silica gel with CH_2_Cl_2_/MeOH (95:5); initial LC/MS purity 51%, *t*_R_ = 1.60 min. ^1^H-NMR (500 MHz, CD_3_OD/CDCl_3_): δ 1.54–1.61 (m, 2H), 1.65–1.78 (m, 5H), 1.80–1.97 (m, 5H), 2.58–2.70 (m, 1H), 2.77 (dd, *J* = 17.9 Hz, *J* = 5.4 Hz, 1H), 2.99–3.21 (m, 4H), 3.36–3.79 (m, 9H), 4.59 (m, 1H), 7.08 (d, *J* = 8.2 Hz, 1H), 7.12 (s, 1H), 7.21 (d, *J* = 7.6 Hz, 1H), 7.41 (t, *J* = 7.9 Hz, 1H), 7.62 (d, *J* = 7.2 Hz, 1H). ^13^C-NMR (500 MHz, CD_3_OD/CDCl_3_): δ 20.1, 24.3, 26.0, 30.3, 35.6, 37.5, 44.9, 46.5, 48.8, 51.7, 56.6, 113.7, 118.2, 119.9, 122.9, 124.0 (q, ^1^*J*_CF_ = 272 Hz), 125.1, 130.1, 132.4, 149.7, 175.2, 176.9. HRMS calcd for [M + H]^+^: C_25_H_34_N_4_O_3_F_3_ *m/z* 495.2578, found 495.2575.

*N-((S)-1-{4-[4-((3-trifluoromethyl)phenyl)piperazin-1-yl]butyl}pyrrolidin-2,5-dion-3-yl)cyclohexanecarboxamide* [**9**{*9,3*}]. Yield: 12 mg (46% isolated yield) as a yellow oil following chromatographic purification over silica gel with CH_2_Cl_2_/MeOH (90:7); initial LC/MS purity 62%, *t*_R_ = 1.67 min.^1^H-NMR (300 MHz, CDCl_3_): *δ* 1.15–1.42 (cluster, 8H), 1.64–1.86 (m, 4H), 2.06-2.18 (m, 3H), 2,56 (br s, 4H), 2.61–2.69 (dd, *J* = 18.2 Hz, *J* = 4.9 Hz, 1H), 2.99–3.12 (q, 1H), 3.27–3.35 (m, 2H), 3.56–3.75 (m, 6H), 4.65–4.73 (m, 1H), 7.06–7.08 (d, *J* = 8.2 Hz, 2H), 7.11 (s, 1H), 7.20–7.24 (m, 1H), 7.38-7.42 (t, *J* = 8.2 Hz, 1H). MS calcd for [M + H]^+^: C_26_H_36_N_4_O_3_F_3_ *m/z* 509.2, found 509.3.

*N-((S)-1-{4-[4-((3-trifluoromethyl)phenyl)piperazin-1-yl]butyl}pyrrolidin-2,5-dion-yl)benzamide* [**9**{*9,4*}]. Yield: 11 mg (44% isolated yield) as a yellow oil following chromatographic purification over silica gel with CH_2_Cl_2_/MeOH (92:8): initial LC/MS purity 40%, *t*_R_ = 1.62 min. ^1^H-NMR (500 MHz, CD_3_OD/CDCl_3_): δ 1.68–1.80 (m, 1H), 1.86–1.99 (m, 3H), 2.98 (dd, *J* = 17.9 Hz, *J* = 5.8 Hz, 1H), 3.07–3.24 (m, 4H), 3.38–3.73 (m, 9H), 4.85 (m, 1H), 7.03 (d, *J* = 8.4 Hz, 1H), 7.06 (s, 1H), 7.21 (d, *J* = 7.6 Hz, 1H), 7.37–7.44 (m, 3H), 7.51 (t, *J* = 7.4 Hz, 1H), 7.88 (d, *J* = 7.5 Hz, 2H), 8.42 (d, *J* = 7.5 Hz, 1H). ^13^C-NMR (500 MHz, CD_3_OD/CDCl_3_): δ 19.9, 24.1, 35.3, 46.4, 50.1, 51.6, 56.4, 118.1, 119.8, 124.1 (q, ^1^*J*_CF_ = 272 Hz), 127.3, 128.5, 129.9, 132.4, 149.6, 175.1. HRMS calcd for [M + H]^+^: C_26_H_30_N_4_O_3_F_3_ *m/z* 503.2265, found 503.2242.

*N-((S)-1-{4-[4-((3-trifluoromethyl)phenyl)piperazin-1-yl]butyl}pyrrolidin-2,5-dion-3-yl)-2-(bicyclo[2.2.1]heptan-2-yl)acetamide* [**9**{*9,5*}]. Yield: 15 mg (57% isolated yield) following chromatographic purification over silica gel with CH_2_Cl_2_/MeOH (90:10): initial LC/MS purity 59%, *t*_R_ = 1.78 min. ^1^H-NMR (CDCl_3_) *δ* 1.07–1.28 (m, 6H), 1.47–1.52 (m, 2H) 1.60 (m, 1H), 1.80–2.01 (m, 4H), 2.06–2.09 (m, 1H), 2.17–2.23 (m, 2H), 2.67–2.76 (s, 1H), 2.97–3.13 (m, 6H), 3.23–3.37 (m, 4H), 3.61–3.69 (m, 4H), 4.65–4.82 (m, 1H,) 7.06–7.12 (m, 2H), 7.20–7.26 (t, 1H), 7.34–7.43 (m, 2H). MS calcd for [M + H]^+^ C_29_H_37_N_4_O_3_F_3_ *m/z* 535.3, found 535.4.

*N-((S)-1-{4-[4-((3-trifluoromethyl)phenyl)piperazin-1-yl]butyl}pyrrolidin-2,5-dion-yl)-1-adamatanecarboxamide* [**9**{*9,6*}]. Yield: 22 mg (78% isolated yield) as a yellow oil following chromatographic purification over silica gel with CH_2_Cl_2_/OH (95:5): Initial LC/MS purity 56%, *t*_R_ = 1.85 min. ^1^H-NMR (500 MHz, CD_3_OD/CDCl_3_): δ 1.61–1.68 (m, 3H), 1.69–1.77 (m, 4H), 1.78–1.95 (m, 9H), 2.01 (s, 3H), 2.78 (dd, *J* = 17.8 Hz, *J* = 5.5 Hz, 1H), 2.99 (dd, *J* = 17.8 Hz, *J* = 9.1 Hz, 1H), 3.04–3.25 (m, 3H), 3.30–3.77 (m, 9H), 4.41 (m, 1H), 7.07 (d, *J* = 8.5 Hz, 1H), 7.11 (s, 1H), 7.19 (d, *J* = 7.7 Hz, 1H), 7.40 (t, *J* = 7.9 Hz, 1H). ^13^C-NMR (500 MHz, CD_3_OD/CDCl_3_): δ 19.9, 24.2, 27.9, 35.1, 36.2, 37.5, 38.8, 40.4, 46.4, 48.9, 51.4, 56.2, 113.5, 117.9, 119.7, 122.8, 123.9 (q, ^1^*J*_CF_ = 272 Hz), 124.9, 129.9, 131.7, 149.7, 175.1, 176.7, 179.1. HRMS calcd for [M + H]^+^: C_30_H_40_N_4_O_3_F_3_ *m/z* 561.3047, found 561.3024.

*1-(Benzoyl)-N-{3-[4-((3-trifluoromethyl)phenyl)piperazin-1-yl]propyl}-L-prolinamide* [**10**{*8,4*}]. Yield 14.5 mg (61% isolated yield) following chromatographic purification over silica gel with CH_2_Cl_2_/MeOH (92:8): Initial LC/MS purity 93%, *t*_R_ = 1.58 min. ^1^H-NMR (300 MHz, CDCl_3_): δ 1.25–1.28 (m, 3H), 1.77–1.85 (m, 2H), 2.02–2.09 (m, 2H), 2.59–2.62 (m, 6H), 3.25 (m, 4H), 3.38–3.40 (m, 2H), 3.60 (m, 1H), 4.65 (t, *J* = 7.5 Hz, 1H), 7.02–7.08 (m, 4H), 7.34 (m, 1H), 7.40–7.42 (d, 4H), 7.53 (br s, 1H). MS calcd for [M + H]^+^: C_26_H_32_N_4_O_2_F_3_ *m/z* 489.2, found 489.6.

*1-(Adamantylcarbonyl)-N-{3-[4-((3-trifluoromethyl)phenyl)piperazin-1-yl]propyl}-L-prolinamide* [**10**{*8,6*}]. Yield: 13 mg (47% isolated yield) following chromatographic purification over silica gel with CH_2_Cl_2_/MeOH (92:8): Initial LC/MS purity 86%, *t*_R_ = 1.90 min. ^1^H-NMR (300 MHz, CDCl_3_): δ 1.25–1.28 (m, 4H), 1.67–1.72 (m, 8H), 1.82–1.95 (m, 2H), 1.99–2.15 (m, 4H), 2.17–2.18 (m, 1H), 2.59 (br s, 4H), 2.65 (br s, 6H), 3.26–3.35 (m, 4H), 3.72–3.83 (m, 2H), 4.56–4.58 (m, 1H), 7.04–7.10 (m, 3H), 7.31–7.32 (t, 1H), 7.34–7.37 (m, 1H). MS calcd for [M + H]^+^: C_30_H_42_N_4_O_2_F_3_ *m/z* 547.3, found 547.4.

*1-(Cyclopentylcarbonyl)-N-{4-[4-((3-trifluoromethyl)phenyl)piperazin-1-yl]butyl}-L-prolinamide* [**10**{*9,2*}]. Yield: 12 mg (49% isolated yield) as a yellow oil following chromatographic purification over silica gel with CH_2_Cl_2_/MeOH (92:8): Initial LC/MS purity 83%, *t*_R_ = 1.60 min. ^1^H-NMR (500 MHz, CDCl_3_): δ 1.54–1.64 (m, 4H), 1.65–1.79 (m, 4H), 1.80–1.89 (m, 4H), 1.91–2.01 (m, 2H), 2.02–2.15 (m, 1H), 2.21–2.29 (m, 1H), 2.77–2.89 (m, 1H), 2.9–3.18 (m, 4H), 3.19–3.35 (m, 3H), 3.37–3.45 (m, 1H), 3.47–3.58 (m, 2H), 3.59–3.78 (m, 4H), 4.47 (dd, *J* = 8.0 Hz, *J* = 3.0 Hz, 1H), 7.06 (dd, *J* = 8.3 Hz, *J* = 2.3 Hz, 1H), 7.11 (m, 1H), 7.17–7.25 (m, 2H), 7.39 (t, *J* = 8.0 Hz, 1H). ^13^C-NMR (500 MHz, CDCl_3_): δ 20.7, 24.9, 26.0, 26.3, 28.2, 29.6, 30.1, 38.0, 42.9, 46.5, 47.5, 51.4, 56.5, 60.2, 113.5, 119.7, 122.9, 124.0 (q, ^1^*J*_CF_ = 272 Hz), 125.1, 129.9, 131.8, 149.8, 172.4, 176.6. HRMS calcd for [M + H]^+^: C_26_H_38_N_4_O_2_F_3_ *m/z* 495.2941, found 495.2936.

*1-(Benzoyl)-N-{4-[4-((3-trifluoromethyl)phenyl)piperazin-1-yl]butyl}-L-prolinamide* [**10**{*9,4*}]. Yield: 14 mg (56% isolated yield) as a yellow oil following chromatographic purification over silica gel with CH_2_Cl_2_/MeOH (92:8): Initial LC/MS purity 89%, *t*_R_ = 1.58 min. ^1^H-NMR (500 MHz, CDCl_3_): δ 1.57–1.71 (m, 2H), 1.79–1.98 (m, 3H), 1.99–2.09 (m, 1H), 2.13–2.30 (m, 2H), 2.69–2.94 (m, 2H), 3.01–3.42 (m, 8H), 3.46–3.62 (m, 3H), 3.67–3.76 (m, 1H), 4.61 (t, *J* = 7.5 Hz, 1H), 6.88–6.99 (m, 2H), 7.17 (d, *J* = 7.7 Hz, 1H), 7.30 (m, 1H), 7.33–7.47 (m, 4H), 7.61 (d, *J* = 6.9 Hz, 2H). ^13^C-NMR (500 MHz, CDCl_3_): δ 20.4, 25.7, 25.9, 28.9, 38.0, 46.2, 50.8, 51.5, 56.5, 60.9, 113.3, 115.4, 117.7, 119.6, 122.9, 124.0 (q, ^1^*J*_CF_ = 272 Hz), 125.1, 127.5, 128.4, 129.8, 130.6, 131.7, 135.9, 149.8, 162.3, 170.5, 172.3. HRMS calcd for [M + H]^+^: C_27_H_34_N_4_O_2_F_3_ *m/z* 503.2628, found 503.2629.

*1-((Bicyclo[2.2.1]heptan-2-yl)acetyl)-N-{4-[4-((3-trifluoromethyl)phenyl)piperazin-1-yl]butyl}-L-prolinamide* [**10**{*9,5*}]. Yield: 15 mg (59% isolated yield) following chromatographic purification over silica gel with CH_2_Cl_2_/MeOH (90:10): Initial LC/MS purity 91%, *t*_R_ = 1.80 min. ^1^H-NMR (300MHz, CDCl_3_) *δ* (ppm) 1.01–1.42 (m, 3H), 1.25–1.32 (m, 5H), 1.79 (m, 1H), 1.93–1.98 (m, 3H), 2.10–2.35 (m, 4H), 2.44 (m, 2H), 2.62 (m, 4H), 3.25 (m, 6H), 3.36–3.45 (m, 3H), 4.54–4.57 (d, *J* = 7.9 Hz, 1H), 7.04–7.10 (m, 3H), 7.31–7.33 (m, 1H), 7.35–7.38 (d, 1H); MS calcd for [M + H]^+^ C_28_H_38_N_4_O_3_F_3_ *m/z* 535.3, found 535.6.

*1-(Adamantylcarbonyl)-N-{4-[4-((3-trifluoromethyl)phenyl)piperazin-1-yl]butyl}-L-prolinamide* [**10**{*9,6*}]. Yield: 13 mg (46% isolated yield) as a yellow oil following chromatographic purification over silica gel with CH_2_Cl_2_/MeOH (92:8): Initial LC/MS purity 86%, *t*_R_ = 1.86 min. ^1^H-NMR (500 MHz, CDCl_3_): δ 1.51–1.63 (m, 2H), 1.65–1.77 (m, 7H), 1.86–2.19 (m, 14H), 2.76–3.17 (m, 4H), 3.18–3.58 (m, 7H), 3.69–3.91 (m, 3H), 4.54 (m, 1H), 6.80 (s, 1H), 7.05 (dd, *J* = 8.3 Hz, *J* = 2.1 Hz, 1H), 7.10 (s, 1H), 7.15 (d, *J* = 7.7 Hz, 1H), 7.37 (t, *J* = 8.0 Hz, 1H). ^13^C-NMR (500 MHz, CD_3_OD/CDCl_3_): δ 14.0, 20.9, 26.5, 28.1, 36.4, 38.1, 41.8, 47.1, 48.5, 51.8, 56.9, 60.4, 62.4, 112.9, 119.3, 122.9, 124.1 (q, ^1^*J*_CF_ = 272 Hz), 125.1, 129.7, 131.6, 150.2, 173.1, 177.1. HRMS calcd for [M + H]^+^: C_31_H_44_N_4_O_2_F_3_ *m/z* 561.3411, found 561.3409.

### 4.5. Solid-Phase Synthesis on BAL-PS Lanterns

*Reductive Amination Protocol.* The Lanterns were divided into two groups and were placed in glass vials containing a suspension of sodium cyanoborohydride ([NaBH_3_CN] = 100 mmol) and the amine ([Diversity reagent **2**] = 250 mmol, Figure 4) in a 1% acetic acid in 3 mL of DMF. The reaction mixture was allowed to stand overnight at 60 °C and was then removed via a drilled adapter. The Lanterns were first washed with 10% AcOH in DMF (1 × 5 min) then with the standard washing protocol, and after allowed to dry in the open air.

#### 4.5.1. Secondary amine acylation protocol

Two DMF solutions (2 mL) containing a Fmoc-protected amino acid and DIC each, were freshly prepared in a standard Schott flask before acylation ([Fmoc-AA-OH] = 200 mmol, [DIC] = 100 mmol), and were left for 10 min to form an active anhydride. Then the Lanterns were immersed in a preactivated solution and left overnight at room temperature. The solution was decanted, and the Lanterns were washed following the standard washing protocol. The acylation was repeated one time more for 4 h.

#### 4.5.2. Standard Fmoc-deprotection protocol

The Fmoc-deprotection step was carried out by immersing the Lanterns in a mixture of piperidine and DMF (20/80, v/v) for 60 min. A 100-mL standard flask, equipped with a drilled topper was used. After removal of the deprotection solution, the Lanterns were washed following the standard washing protocol.

#### 4.5.3. Standard acylation with carboxylic acids

Six DMF solutions (2 mL), containing carboxylic acid (Figure 5: Diversity reagent **6**), HBTU and DIEA each, were freshly prepared in a standard Schott flask before coupling ([R_2_-CO_2_H] = 120 mmol; [HBTU] = 120 mmol; [DIEA] = 240 mmol). The Lanterns were immersed for 2 h in the coupling solution at a room temperature. The solution was decanted, and the Lanterns were washed following the standard washing procedure. The procedure described above was repeated.

#### 4.5.4. Cleavage/cyclization protocol for succinimide derivatives formation

Chemsets **7a** and **7b** were placed in glass vials containing a mixture of TFA/CHCl_3_/SOCl_2_ (1 mL, 50/50/1.5, v/v/v). The reaction was allowed to stand for 10 hours at 40 °C. Afterwards, the reaction solution was removed under nitrogen flow. A 100 µL portion of acetonitrile/water (50/50, v/v) containing a 0.1% TFA was poured into each tube to dissolve the samples. The samples were then frozen at −80 °C and lyophilized. 

#### 4.5.5. Cleavage of the proline derivatives off the lantern

A 500 µL aliquot of TFA was dispensed into individual glass tubes. Cleavage was carried out for 60 min. The cleavage cocktail was removed from the tubes under nitrogen flow. A 100 µL portion of acetonitrile/water (50/50, v/v) containing a 0.1% TFA was poured into each tube to dissolve the sample. Then the samples were frozen at −80 °C and lyophilized.

### 4.6. Analytical Data for 12 Library Members Synthesized on SynPhase Lanterns

*N-((S)-1-{3-[4-((3-trifluoromethyl)phenyl)piperazin-1-yl]propyl}pyrrolidin-2,5-dion-yl)cyclohexanecarbox-amide* [**9**{*8,3*}]. Yield: 5.1 mg (47%) as a yellow oil following chromatographic purification over silica gel with CH_2_Cl_2_/MeOH (90:10); initial LC/MS purity 74%, *t*_R_ = 1.66 min. MS calcd for [M + H]^+^: C_25_H_34_N_4_O_3_F_3_ *m/z* 495.2, found 495.1.

*N-((S)-1-{3-[4-((3-trifluoromethyl)phenyl)piperazin-1-yl]propyl}pyrrolidin-2,5-dion-yl)benzamide* [**9**{*8,4*}]. Yield: 3.8 mg (35%) as a yellow oil following chromatographic purification over silica gel with CH_2_Cl_2_/MeOH (90:10); initial LC/MS purity 70%, *t*_R_ = 1.60 min. MS calcd for [M + H]^+^: C_25_H_28_N_4_O_3_F_3_ *m/z* 489.2, found 489.1.

*N-((S)-1-{3-[4-((3-trifluoromethyl)phenyl)piperazin-1-yl]propyl}pyrrolidin-2,5-dion-yl)-2-(bicyclo[2.2.1]heptan-2-yl)acetamide* [**9**{*8,5*}]. Yield: 5.1 mg (45%) as a yellow oil following chromatographic purification over silica gel with CH_2_Cl_2_/MeOH (90:10); initial LC/MS purity 77%, *t*_R_ = 1.78 min. MS calcd for [M + H]^+^: C_27_H_36_N_4_O_3_F_3_ *m/z* 521.2, found 521.4.

*N-((S)-1-{4-[4-((3-trifluoromethyl)phenyl)piperazin-1-yl]butyl}pyrrolidin-2,5-dion-3-yl)cyclohexane-carboxamide* [**9**{*9,3*}]. Yield: 5.4 mg (48% isolated yield) as a yellow oil following chromatographic purification over silica gel with CH_2_Cl_2_/MeOH (90:10); initial LC/MS purity 68%, *t*_R_ = 1.67 min. MS calcd for [M + H]^+^: C_26_H_36_N_4_O_3_F_3_ *m/z* 509.2, found 509.4.

*N-((S)-1-{4-[4-((3-trifluoromethyl)phenyl)piperazin-1-yl]butyl}pyrrolidin-2,5-dion-yl)benzamide* [**9**{*9,4*}]. Yield: 4.9 mg (44% isolated yield) as a yellow oil following chromatographic purification over silica gel with CH_2_Cl_2_/MeOH (90:10); initial LC/MS purity 64%, *t*_R_ = 1.62 min. MS calcd for [M + H]^+^: C_26_H_30_N_4_O_3_F_3_ *m/z* 503.2, found 503.3.

*N-((S)-1-{4-[4-((3-trifluoromethyl)phenyl)piperazin-1-yl]butyl}pyrrolidin-2,5-dion-yl)-1-adamatanecarboxamide* [**9**{*9,6*}]. Yield: 5.6 mg (46% isolated yield) as a yellow oil following chromatographic purification over silica gel with CH_2_Cl_2_/MeOH (90:10); initial LC/MS purity 75%, *t*_R_ = 1.85 min. MS calcd for [M + H]^+^: C_30_H_40_N_4_O_3_F_3_ *m/z* 561.3, found 561.4.

*1-(Cyclohexylcarbonyl)-N-{3-[4-((3-trifluoromethyl)phenyl)piperazin-1-yl]propyl}-L-prolinamide* [**10**{*8,3*}]. Yield: 6.3 mg as a yellow oil (58% isolated yield) following chromatographic purification over silica gel with CH_2_Cl_2_/MeOH (90:10); initial LC/MS purity 96%, *t*_R_ = 1.69 min. MS calcd for [M + H]^+^: C_26_H_38_N_4_O_2_F_3_ *m/z* 495.3, found 495.4.

*1-(Benzoyl)-N-{3-[4-((3-trifluoromethyl)phenyl)piperazin-1-yl]propyl}-L-prolinamide* [**10**{*8,4*}]. Yield: 5.1 mg as a yellow oil (47% isolated yield) following chromatographic purification over silica gel with CH_2_Cl_2_/MeOH (90:10); initial LC/MS purity 94%, *t*_R_ = 1.58 min. MS calcd for [M + H]^+^: C_26_H_32_N_4_O_2_F_3_ *m/z* 489.2, found 489.4.

*1-((Bicyclo[2.2.1]heptan-2-yl)acetyl)-N-{3-[4-((3-trifluoromethyl)phenyl)piperazin-1-yl]propyl}-L-prolinamide* [**10**{*8,5*}]. Yield: 6.3 mg as a yellow oil (55% isolated yield) following chromatographic purification over silica gel with CH_2_Cl_2_/MeOH (90:10); initial LC/MS purity 96%, *t*_R_ = 1.81 min; MS calcd for [M + H]^+^ C_28_H_40_N_4_O_2_F_3_ *m/z* 521.3, found 521.5.

*1-(Cyclohexylcarbonyl)-N-{4-[4-((3-trifluoromethyl)phenyl)piperazin-1-yl]butyl}-L-prolinamide* [**10**{*9,3*}]. Yield: 5.5 mg as a yellow oil (49% isolated yield) following chromatographic purification over silica gel with CH_2_Cl_2_/MeOH (90:10); initial LC/MS purity 97%, *t*_R_ = 1.68 min. MS calcd for [M + H]^+^: C_27_H_40_N_4_O_2_F_3_ *m/z* 509.3, found 509.2.

*1-(Benzoyl)-N-{4-[4-((3-trifluoromethyl)phenyl)piperazin-1-yl]butyl}-L-prolinamide* [**10**{*9,4*}]. Yield: 6.2 mg as a yellow oil (56% isolated yield) following chromatographic purification over silica gel with CH_2_Cl_2_/MeOH (90:10); initial LC/MS purity 98%, *t*_R_ = 1.57 min. MS calcd for [M + H]^+^: C_27_H_34_N_4_O_2_F_3_ *m/z* 503.2, found 503.3.

*1-(Adamantylcarbonyl)-N-{4-[4-((3-trifluoromethyl)phenyl)piperazin-1-yl]butyl}-L-prolinamide* [**10**{*9,6*}]. Yield: 6.5 mg as a yellow oil (54% isolated yield) following chromatographic purification over silica gel with CH_2_Cl_2_/MeOH (90:10); initial LC/MS purity 98%, *t*_R_ = 1.85 min. MS calcd for [M + H]^+^: C_31_H_44_N_4_O_2_F_3_ *m/z* 561.3, found 561.4.

Radioligand binding studies

The *in vitro* affinity for native serotonin 5-HT_1A_ and 5-HT_2A_ receptors was determined by inhibiting [3H]-8-OH-DPAT (17 Ci/mmol; NEN Chemicals) and [^3^H]-ketanserin (88 Ci/mmol; NEN Chemicals) binding to rat hippocampal and cortical membranes, respectively. Membrane preparation and a general assay procedure were carried out according to the previously published protocols [18]. Two compound concentrations were tested: 0.1 and 1 μM, each run in triplicate. The *K*_i_ values, estimated on the basis of three independent binding experiments SEM ≤ 22%.

## 5. Conclusions

In summary, we have successfully adapted a previous synthetic protocol to enable the synthesis of new chemical entities within the Distributed Drug Discovery (D3) project. As an outcome of student involvement we have disclosed a 24-member library of novel arylpiperazines, obtained on BAL-PS-SynPhase Lanterns and on BAL-MBHA-PS resin. A subset of these was then tested on 5-HT_1A_ and 5-HT_2A_ receptors as potential agents for the treatment of CNS-directed disorders. The biological results indicated that introduction of the *m*-CF_3_ substituent in the phenylpiperazine fragments opens the possibility of obtaining dual 5-HT_1A_/5-HT_2A_ agents. While the biological testing is centralized, this combination of distributed synthesis with screening will enable a D3 network of students worldwide to participate, as part of their education, in the synthesis and testing of this class of biologically active compounds.
